# Analysis of surgical strategies and efficacy in the treatment of Os odontoideum with atlantoaxial dislocation

**DOI:** 10.1186/s13018-023-03517-x

**Published:** 2023-01-13

**Authors:** Baohui Yang, Teng Lu, Xijing He, Haopeng Li

**Affiliations:** Department of Orthopedics, Second Affiliated Hospital of Xi’an Jiaotong University, Xi’an , Shaanxi Province China

**Keywords:** Os odontoideum, Atlantoaxial dislocation, Reduction, Posterior fusion, Cervical

## Abstract

**Background:**

There are many classification systems for atlantoaxial dislocation (AAD). Among these systems, the definitions of irreducible AAD remain vague, and its treatments are not unified.

**Objective:**

To explore the surgical strategies and efficacy for the treatment of os odontoideum (OO) with AAD.

**Methods:**

The clinical data of 56 OO patients with AAD who underwent surgery from January 2017 to June 2021 were retrospectively analyzed. AAD was classified into four types, Type I and type II were treated with posterior fixation and fusion. Type III received posterior fixation and fusion after irreducible dislocations were converted to reducible dislocations by translateral mass release or transoral release. Type IV required transoral release for conversion into reducible dislocations before posterior fixation and fusion. The operation time, blood loss, and complications were recorded. The preoperative and postoperative neurological function changes were assessed using the Japanese Orthopedic Association (JOA) score. Postoperative fusion status was assessed by X-ray.

**Results:**

There were 40 cases of type I-II, 14 cases of type III, and two cases of type IV AAD. The operation times of single posterior fixation and fusion, combined translateral mass release and combined transoral release were 130.52 ± 37.12 min, 151.11 ± 16.91 min and 188.57 ± 44.13 min, the blood loss were 162.63 ± 58.27 mL, 235.56 ± 59.94 mL, 414.29 ± 33.91 mL, respectively. One patient with type III died, one with type III underwent revision surgery due to infection, and three patients with type I had further neurological deterioration after operation. fifty-five patients were followed up for 12–24 months. The follow-up results showed that enough decompression was achieved and that fixation and fusion were effective. The JOA score increased from 9.58 ± 1.84 points preoperative to 13.09 ± 2.68 points at 3 months after operation, 14.07 ± 2.83 points at 6 months and 14.25 ± 2.34 at 12 months after operation, all significant differences compared with preoperative results (*P* < 0.05).

**Conclusion:**

OO patients with irreducible AAD can be treated by translateral mass release or transoral release combined with posterior fixation and fusion, while some of those with bony fusion can be treated by transoral release combined with posterior fixation and fusion.

## Introduction

Os odontoideum (OO) is a rare cervical anomaly, mainly including ossicles isolated from the fractured odontoid processes of the axis, which can be mainly attributed to trauma or congenital factors [1, 2]. Asymptomatic patients and those without instability can undergo surveillance clinical and imaging examinations [3]. The lack of bony connection between the axis and the odontoid process can leads to atlantoaxial instability, and the long-term instability will trigger atlantoaxial dislocation (AAD), resulting in high cervical spinal cord compression and chronic upper motor neuron injury. In some patients, AAD complicated with acute injury can lead to death [4]. Therefore, it is widely accepted that OO patients with atlantoaxial instability or AAD should receive surgical treatment [5] to stabilize the upper cervical spine, to relieve spinal cord compression, and to avoid future neurological injury [6].

There are many classification systems for AAD. Among these systems, the definitions of atlantoaxial instability and reducible AAD are relatively unified, and the therapeutic regimens are also consistent. As techniques have advanced, some cases classified as the irreducible AAD could be converted to the reducible type through anterior release. The definitions of irreducible and AAD are therefore malleable and its treatments are not unified. They include simple posterior fixation and fusion [7] and posterior fixation and fusion surgery following transoral release [8, 9]. OO with AAD has been rarely reported [7, 8] and has not been systematically studied.

In the present study, we thought the classification system of Wang [10] could provide better guidance in clinical practice. First of all, this classification was conducted on the basis of the number of 904 patients, indicating a large number of patients; at the same time, the classification is more comprehensive, which has great guiding significance for the formulation of the surgical plan. Secondly, through the description of the author, it also verifies the good curative effect and few complications. Based on the classification system proposed by Wang [10], we adopted targeted surgical strategies, made recommendations for each patient, and analyzed the efficacy and perioperative complications.

## Materials and methods

### General data

A total of 32 males and 24 females aged 49.36 ± 13.98 years were enrolled, whose course of disease was 8.9 ± 3.38 months (Table 1). Inclusion criteria included: (1) Computed tomography (CT) coronal reconstruction presented the OO, and anterior atlanto-dental interval (ADI) > 3 mm in lateral X-ray and (2) those with acute or chronic clinical manifestation as upper motor neuron injury clinical manifestation of upper motor neuron injury. Exclusion criteria included: (1) AAD caused by infection, tumor, or tuberculosis, (2) coexisting basilar invagination, and (3) history of atlantoaxial surgery or surgery in surrounding areas.

**Table 1:** Basic data of patients

Variable	Data
Age (years)	49.36 ± 13.98
*Sex*	56
Male	32
Female	24
*Etiology*	
Congenital	47
Traumatic	9
*Classification (n)*	
I (instability)	30
II (reducible dislocation)	10
III (irreducible dislocation)	14
IV (bony dislocation)	2
Course of disease (month)	8.91 ± 3.38

#### Etiology and classification

Forty-seven cases of OO were caused by congenital malformation or dysplasia, and nine cases were caused by an old fracture trauma. Coexisting pathologies included rheumatoid arthritis (7), ankylosing spondylitis (1), Kaschin Beck Disease (1), and Down’s Syndrome (1). Preoperative cervical anteroposterior and lateral and dynamic radiography, cervical computed tomography (CT) scan with three-dimensional reconstruction, and cervical magnetic resonance imaging (MRI) were performed on all patients to analyze dislocation, reduction, and spinal cord compression status. Based on the classification criteria proposed by Wang [10], AAD was classified into type I (instability: successful reduction by hyperextension/hyperflexion confirmed by dynamic radiographs), type II (reducible dislocation: reducible by high-weight skull traction under anesthesia), type III (irreducible dislocation: irreducible by high-weight skull traction under anesthesia), and type IV (bony dislocation: C1-C2 bony fusion).

### Ethical review

The study was in accordance with the Declaration of Helsinki and was approved by the Ethics.

Committee of The Second Affiliated Hospital of Xi’an Jiaotong University (Xi’an, China). Written informed consent was obtained from all patients.

### Surgical methods

All the operations were performed by the corresponding author. Except for type I cases, all cases were treated with post anesthesia skull traction with a maximum weight of 1/6 of body weight. Type I and II cases were treated with posterior fixation and fusion. Type III cases received posterior fixation and fusion after irreducible dislocations were converted to reducible dislocations by trans lateral mass release or transoral release. Type IV cases required transoral release for conversion into reducible dislocations before posterior fixation and fusion. Here, the posterior side of the lateral atlantoaxial joint was exposed for patients who underwent trans lateral mass release, the vessels and nerve bundles between C1 and C2 were carefully protected, The C1 and C2 lateral mass joint capsule were incised, The loosening of the joints was completed by the axial distraction of the joints with a 7-mm-wide bone inserted knife into the facet joints, The atlas was levered by the bone knife, and the axis was pushed to reduce AAD; this procedure was monitored through fluoroscopy. After lateral mass joint release, direct posterior fixation and fusion were then performed. In patients with transoral release, Initially, the patient was placed supine, The transoral approach was performed with the assistance the mouth retractor, which could expand the mouth and exposed the posterior wall of the pharynx. The posterior pharyngeal wall layer was longitudinally incised along the middle line. Then, the anterior atlas arch, the axis vertebral body, and the lateral mass joints were gradually exposed toward bilateral, cephalic, and caudal sides. The contractural muscles, the anterior longitudinal ligament, and joint capsules between the atlantoaxial joints were incised and released, and the osteophytes and scar tissues between C1 and the C2 vertebral body, and the apical and alar ligaments were cleaned. The reduction of AAD was then performed by traction and leverage. Then, turn over, take the prone position, Posterior fixation and fusion in the end was then performed. The pedicle screw is the most effective and commonly used fixation technique for the C2 vertebra, for this reason, the pedicle screw was used in most of our cases. But in the case of the high-riding vertebral artery, the pedicle was forced insertion of a screws, which easily damages the medial spinal cord and the lateral vertebral artery (VA), laminar screw was used for fixation in this case.

### Follow-up and observation indicators

The patients were followed up for 12–24 months (13 months on average). The operation time, intraoperative blood loss, and perioperative complications were recorded. The neurological function was assessed with the 17-point Japanese Orthopedic Association (JOA) scoring system, which addresses upper- (4 points) and lower-extremity motor function (4 points), upper- (2 points) and lower-extremity sensory function (2 points), trunk sensory function (2 points), and bladder function (3 points). Other indicators were evaluated by imaging. In detail, X-ray or CT of the cervical spine was conducted at 3 and 6 months after operation, from which the anatomical relationship of atlantoaxial vertebra and the callus of bone graft and healing status were observed. In order to understand whether there is still spinal cord compression after surgery, some patients were reexamined by MRI.

### Statistical processing

SPSS19.0 was used for statistical analysis. JOA scores before and after operation are presented as mean ± standard deviation (${\overline{\text{x}}}$ ± s), and one way ANOVA analysis was used to compare them *P* < 0.05 denoted a statistically significant difference.

## Results

**Fig. 1:**
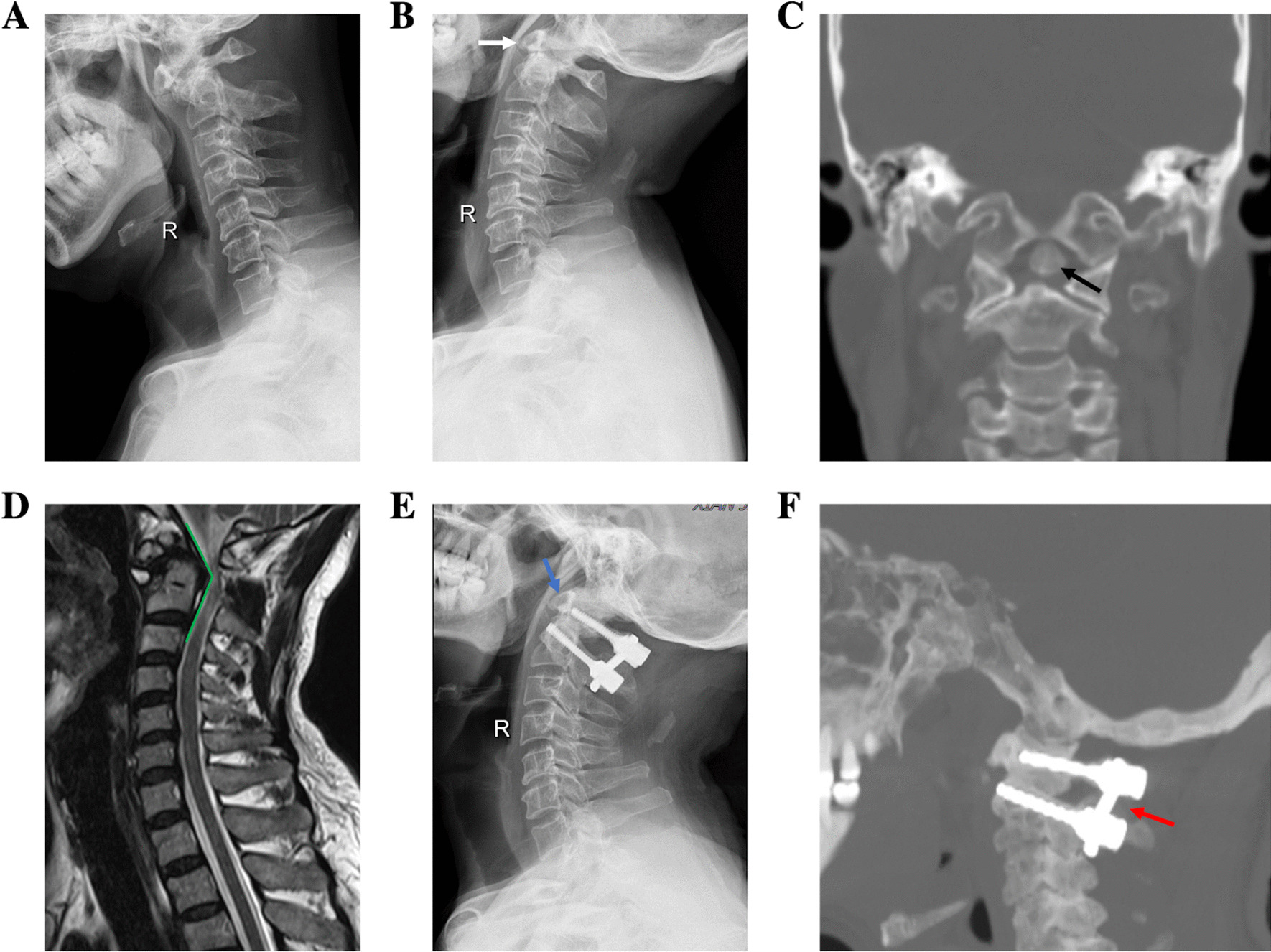
A 50-year-old male patient was admitted for numbness of limbs with walking instability for 6 months, aggravated for 2 months. The ADI was 5.83 mm in preoperative hyperflexion X-ray (**a**), but the atlantoaxial dislocation could be completely reduced in the hyperextension X-ray (**b**, white arrow), So it was classified as type I. Computed tomography (CT) coronal reconstruction presented the OO (**c**, black arrow). The upper cervical spinal cord was compressed by the superior posterior part of axis based on magnetic resonance imaging (MRI), with a cervicomedullary angle of 143° (**d**). Posterior fixation and fusion were performed. Postoperative X-ray showed atlantoaxial fixation by screw and rod, and anatomical reduction was observed (**e**, blue arrow). At 6 months after operation, CT reexamination showed posterior bone graft healing (**f**, red arrow)

**Fig. 2:**
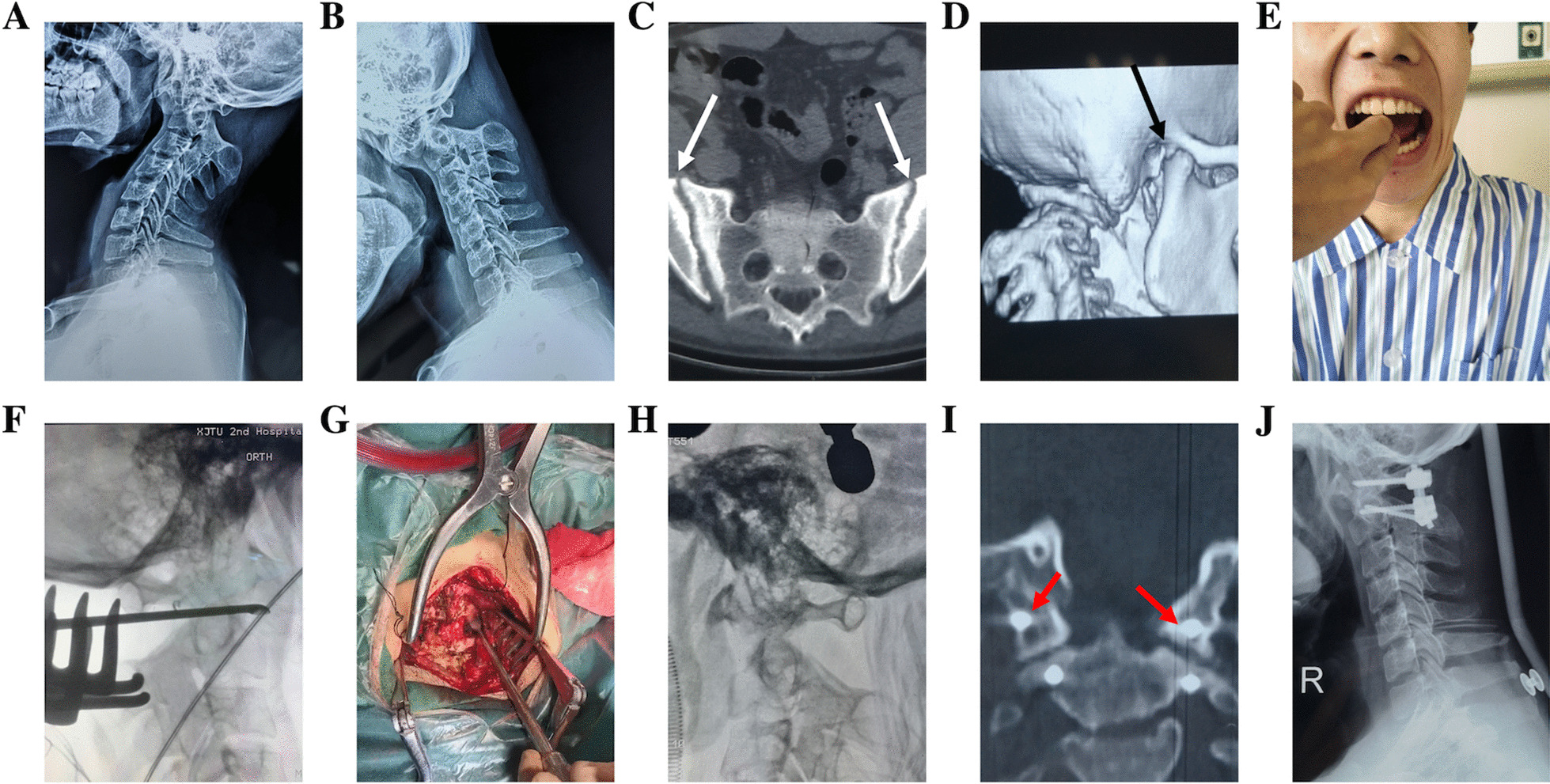
A 23-year-old male patient was admitted for neck pain with numbness of both upper limbs for 8 months. The ADI was 7.41 mm (**a**) on preoperative hyperflexion X-ray and 5.32 mm (**b**) on hyperextension X-ray, and dynamic radiographs showed failed reduction of atlantoaxial dislocation. The patient had ankylosing spondylitis (**c**, white arrow) and difficulty opening his mouth (**e**) due to temporomandibular joint fusion (**d**, black arrow). After anesthesia, the atlantoaxial dislocation could not be completely reduced by skull traction with a weight of 1/6 of body weight, but reduction exceeding 50% was classified as type III. which was converted to type II by translateral mass release (**f**–**h**), followed by posterior fixation and fusion. After operation, the lateral mass was completely released (**i**, red arrow). Postoperative lateral X-rays of the cervical spine showed effective reduction and fixation (**j**)

**Fig. 3:**
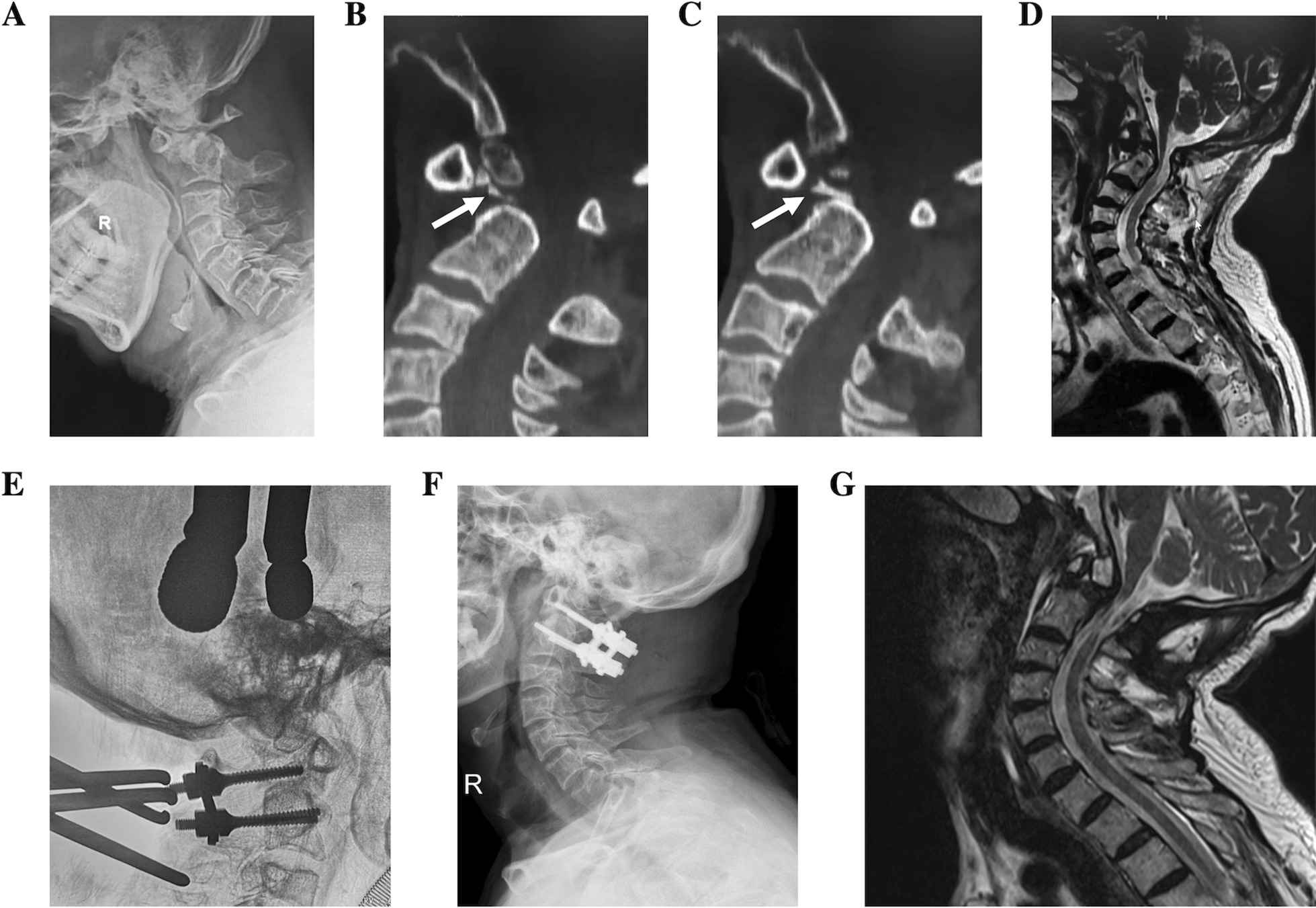
A 56-year-old male patient was admitted for numbness of the right limbs with walking instability for 11 months. Preoperative lateral X-ray showed OO with AAD (**a**), and some discontinuous bony structures were seen between the anterior arches of the atlas and the axis in computed tomography (CT) sagittal reconstruction (**b**, **c**, white arrow). Magnetic resonance imaging (MRI) showed compression of cervicomedullary junction (CMJ) (**d**). Reduction of the atlantoaxial dislocation by intraoperative high-weight skull traction failed, and reduction less than 50% was classified as type III. Following transoral release and posterior fixation and fusion, satisfactory reduction were achieved (**e**, **f**), and MRI showed the elimination of CMJ compression postoperatively (**g**)

**Fig. 4:**
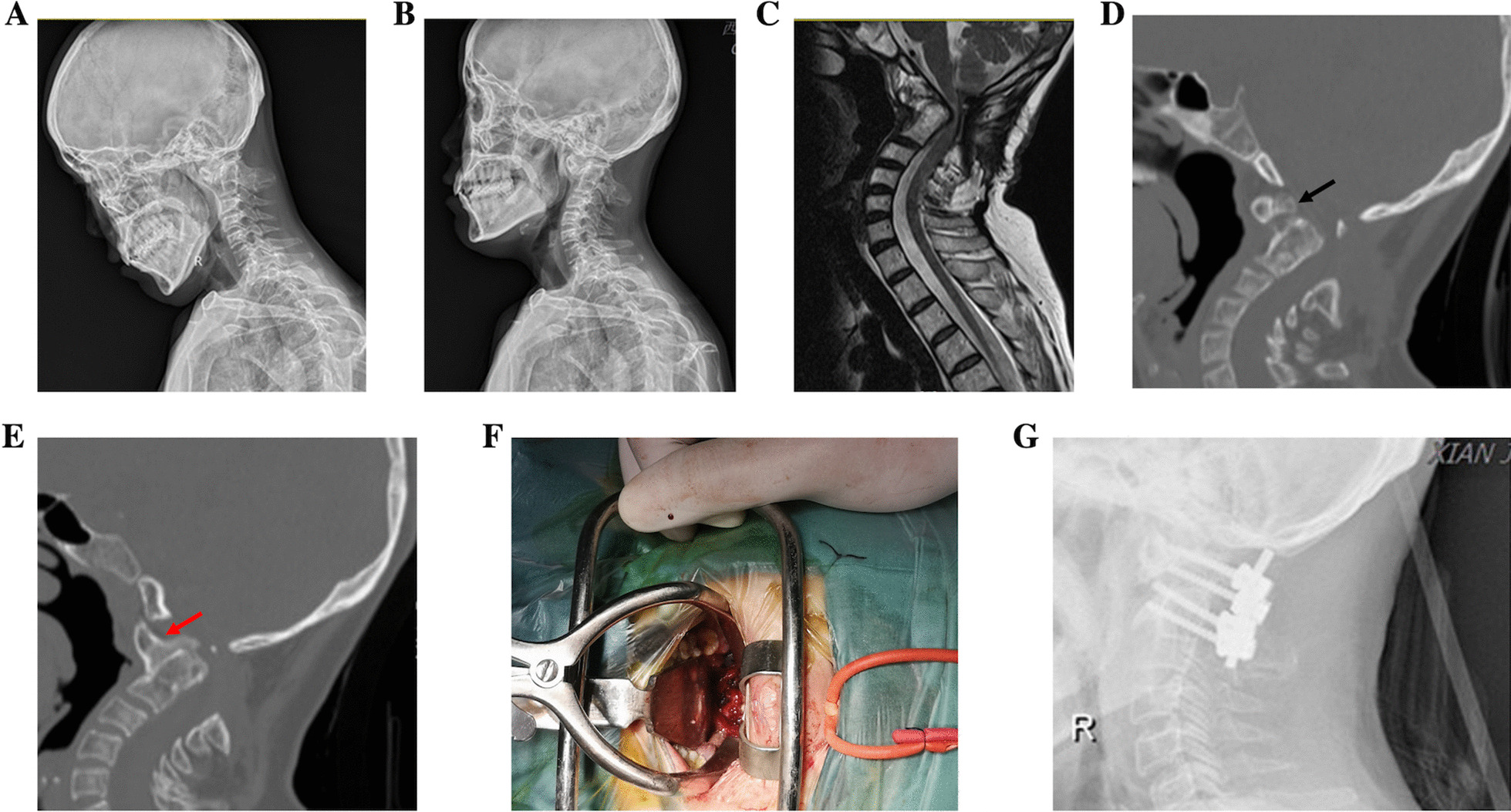
A male patient was admitted for numbness and weakness of limbs for 7 months and rheumatoid arthritis for 5 years. Hyperextension and hyperflexation X-ray presented AAD, and ADI were almost unchanged in dynamic position (**a**, **b**). CT showed OO (**c**, black arrow) and bony fusion between the atlantoaxial joints (**d**, red arrow), so it was classified as type IV. MRI showed compression of the cervicomedullary junction (CMJ) (**e**), followed by transoral release (**f**) and resection of bony fusion tissue. Finally, posterior fixation and fusion were accomplished, and postoperative imaging data showed effective reduction, fixation, and fusion (**g**)

Posterior fixation and fusion were conducted on 30 cases of type I and 10 cases of type II (Fig. 1). In type III cases, irreducible dislocations were converted to reducible dislocations by trans lateral mass release (*n* = 9, Fig. 2) and by transoral release (*n* = 4, Fig. 3), followed by posterior fixation and fusion, respectively. One patient died due to vertebral artery injury in transoral release. In two type IV cases, posterior fixation and fusion were performed after bony dislocations were converted to reducible dislocations by transoral bony decompression and release (Fig. 4). In this group of cases, 55 cases were chronic compression of the spinal cord, and 1 case suffered acute spinal cord injury after a fall on the basis of the original atlantoaxial dislocation. The operation times of single posterior fixation and fusion, combined translateral mass release and combined transoral release were 130.52 ± 37.12 min, 151.11 ± 16.91 min and 188.57 ± 44.13 min, the blood loss were 162.63 ± 58.27 mL, 235.56 ± 59.94 mL, 414.29 ± 33.91 mL, respectively (Table 2).

**Table 2:** Operative characteristics of patients

Surgical approach	Classification	Number of patients	Operation time (min)	Blood loss (mL)
Posterior fixation and fusion	Type I, Type II	40	130.5 ± 37.12	162.63 ± 58.27
Translateral mass release + posterior fixation and fusion	Type III	9	151.1 ± 16.91	235.56 ± 59.94
Transoral release + posterior fixation and fusion	Type III, Type IV	7	188.5 ± 44.13	414.29 ± 33.91

**Fig. 5:**
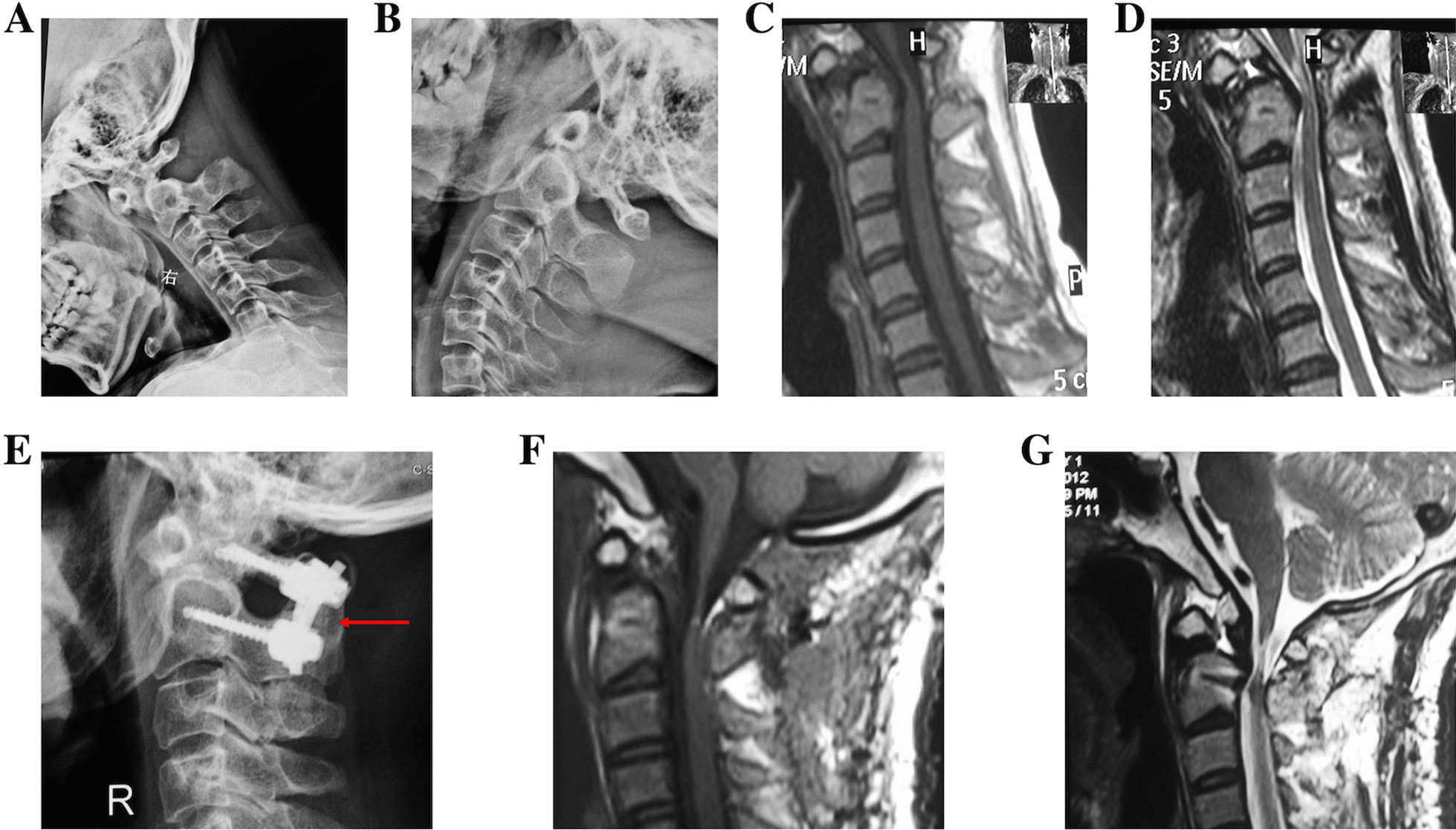
A 45-year-old male patient was admitted for numbness of limbs and walking instability for 2 years. Preoperative dynamic radiographs (**a**, **b**) showed successful reduction of the atlantoaxial dislocation in a hyperextended position, so it was classified as type I. Preoperative T1-weighted MRI showed atrophy of the spinal cord (**c**), and T2-weighted MRI showed high signal intensity of the spinal cord (**d**). Following posterior fixation and fusion, satisfactory reduction and internal fixation were achieved (**e**, red arrow). Postoperative MRI showed that spinal cord compression was relieved (**f**, **g**), and the patients had further neurological deterioration within 6 months after operation

Fifty-five patients were followed up for 12–24 months (13 months on average), in whom the bony fusion rate was 100%, the criteria for fusion were the presence of a continuous trabecular connection in the graft area visible on X-ray or CT [11]. The JOA score increased from 9.58 ± 1.84 points before operation to 13.09 ± 2.68 points at 3 months after operation, 14.07 ± 2.83 points at 6 months and 14.25 ± 2.34 at 12 months after operation, all significant differences comparison with preoperative results (*P* < 0.05). The overall JOA score improved, but the neurological function further deteriorated in three patients with type I after operation (Fig. 5).

**Fig. 6:**
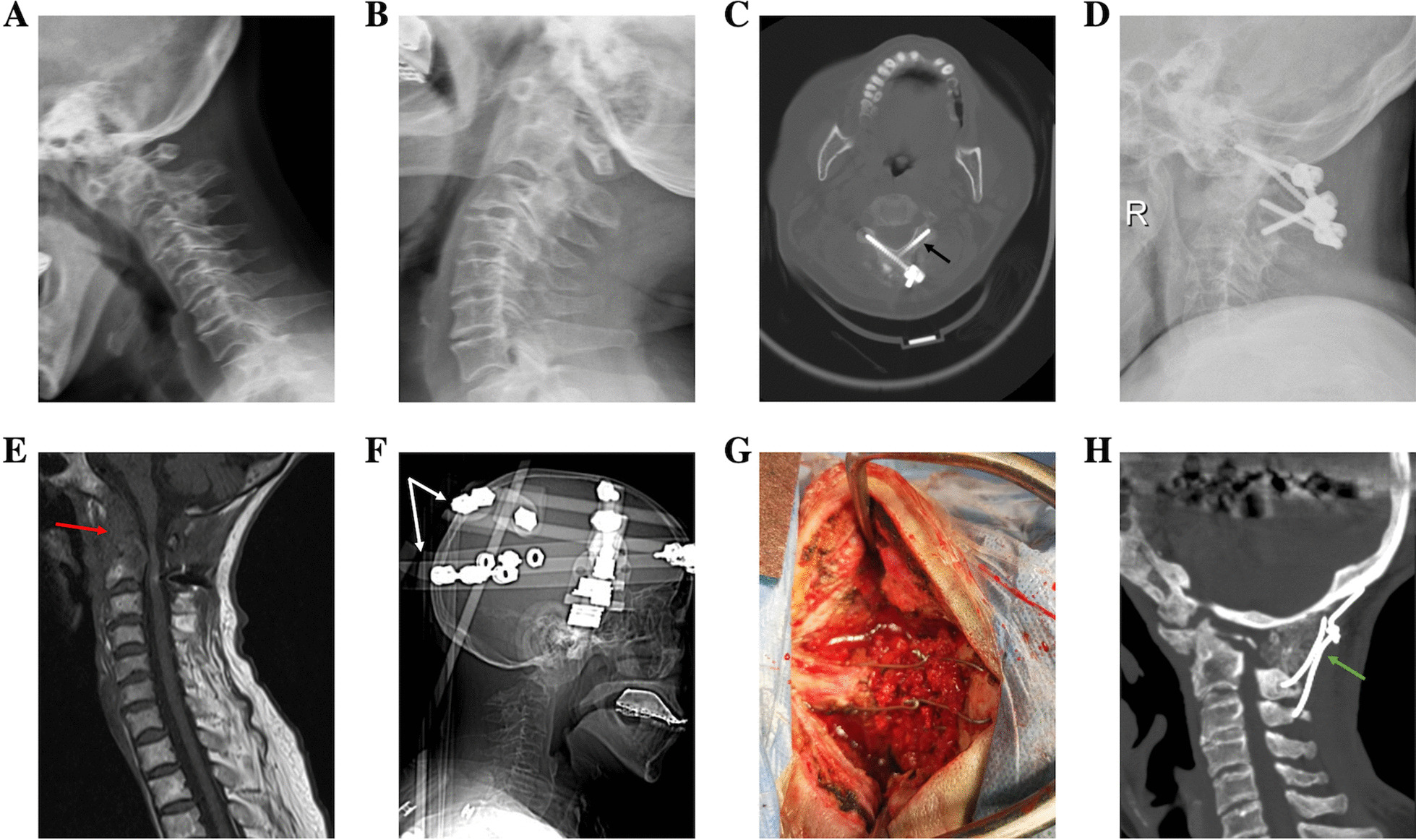
A 64-year-old female patient was admitted for weakness of both legs and walking instability for 9 months. Preoperative dynamic radiographs showed failed reduction of the atlantoaxial dislocation in a hyperextended or hyperflexed position (**a**, **b**), and after intraoperative traction, the atlantoaxial dislocation could still not be reduced, so it was classified as type III. Due to the high-riding vertebral artery, laminar screw fixation of the axis was performed (**c**, black arrow). At 2 months after operation, infection occurred (**e**, red arrow), and the internal fixation loosened and dislocated again (**d**). Debridement and anti-infection treatment were performed, the internal fixation was removed, and the head-ring brace was fixed (**f**, white arrow). Due to loosening of the original screw track, occipitocervical fusion was performed after infection control (**g**). The internal fixation position was acceptable at 3 months after operation, and bone graft healing was almost achieved (**h**, green arrow)

Vertebral artery injury occurred in one patient during transoral release, who eventually died of multiple-organ failure despite trans arterial embolization. Postoperative infection was found in one patient at 2 months after operation. This patient initially presented with occipitocervical radiation pain after surgery, without underlying diseases such as diabetes, so we considered local blocking therapy for great occipital neuralgia, but the symptoms did not significantly relieve. Two months after the operation, CT review showed that the internal fixation was loose, so we had to conduct surgical exploration. After opening the wound, deep pus was found, which was later confirmed as coprococcus by bacterial culture. Then the internal fixation was removed, and anti-infection, debridement, lavage, drainage, and halo external fixation were used to control infection. Finally, the infection was cured following occipitocervical fusion (Fig. 6).

## Discussion

### Characteristics of OO with AAD

In this study, type I and type II cases [*n* = 40 (71%)] were in the majority, suggesting that OO with AAD is mostly reducible. Type III and type IV cases [*n* = 16 (29%)] were often associated with rheumatoid arthritis, ankylosing spondylitis, and other diseases.

OO is mostly caused by trauma or congenital factors. Due to the lack of bony connection between the axis and the odontoid process, atlantoaxial instability and AAD can occur. Posterior fixation and fusion need to be individually selected, and fixation can usually be limited to the C1-2 level, which can preserve the cervical range of motion to the maximum extent. Occipitocervical fusion was rarely performed for OO with AAD (*n* = 1) in this study.

Previously, posterior atlantoaxial arthrodesis was performed with fixation of the posterior arch of atlas, and the surgical procedures included Gallie steel wire fixation of the posterior arch of atlas and the spinous process of the axis, Brooks wire fixation, and Halifax clamp fixation [12, 13]. However, an intact posterior structure is required for these methods, and the reconstruction effect of the atlantoaxial stability is unsatisfactory [14]. With the development of the pedicle screw technique, firm fixation has been achieved for AAD, and this technique also applies to most other patients [13, 15]. In the cases of high-riding vertebral artery or narrow vertebral pedicle, however, laminar screws are also an alternative, though they have poorer biomechanical stability than pedicle screws [16]. In this study, posterior C1-2 pedicle screw or lateral mass fixation was most often performed.

### Classification

There are many classification methods for AAD, including an etiology- or dislocation direction–based method [17] that is not effective at guiding clinical treatment. In 1968, Greenberg [18] first classified AAD into two subtypes (reducible and irreducible), based on which corresponding therapeutic strategies were put forward. This classification method was a milestone, but it was too simple to fully guide the clinical approach. In 2003, based on the reduction status after skull traction and transoral anterior release, Zhu et al. [19] classified AAD into reducible dislocation, hard-to-reduce dislocation, and irreducible dislocation. This classification proved to have high practical value. As techniques have advanced, however, some cases classified as the irreducible type in the above way could be converted to the reducible type through anterior release, making Yin's classification method no longer clear [20]. In 2013, Wang [10] classified AAD into instability, reducible dislocation, irreducible dislocation, and bony dislocation and put forward corresponding therapeutic strategies: Type III cases are treated by posterior fixation and fusion after irreducible dislocations are converted to reducible dislocations by transoral release, while type IV cases can be treated by odontoidectomy. In this study, however, posterior fixation and fusion were conducted on nine cases of type III AAD following conversion to type II by trans lateral mass release (Fig. 2), four cases of type III following transoral release (Fig. 3), and two cases of type IV following transoral bony decompression and release (Fig. 4). Similar to the classification method of Wang [10], a new classification method for AAD was proposed by Tan et al. [21], which can also guide clinical practice well. However, transoral release followed by posterior fixation and fusion is also recommended for type 0 (irreducible after traction), excluding the simple posterior fixation and fusion followed by trans lateral mass release described herein. Therefore, we believe that these classification methods used for guiding therapeutic strategies can be further improved.

### Surgical strategies

Effective skull traction for atlantoaxial reduction is an important way to simplify surgery and reduce complications. Except for type I cases, all cases here were subjected to postanesthesia skull traction with a maximum weight of 1/6 of body weight. The muscles, ligaments, and joint capsules blocking atlantoaxial reduction could be relaxed by traction, benefitting intraoperative reduction. The results showed that satisfactory reduction was achieved by traction in type II, and reduction exceeding 50% was achieved in nine cases of type III. Therefore, postanesthesia traction can be recommended.

**Fig. 7:**
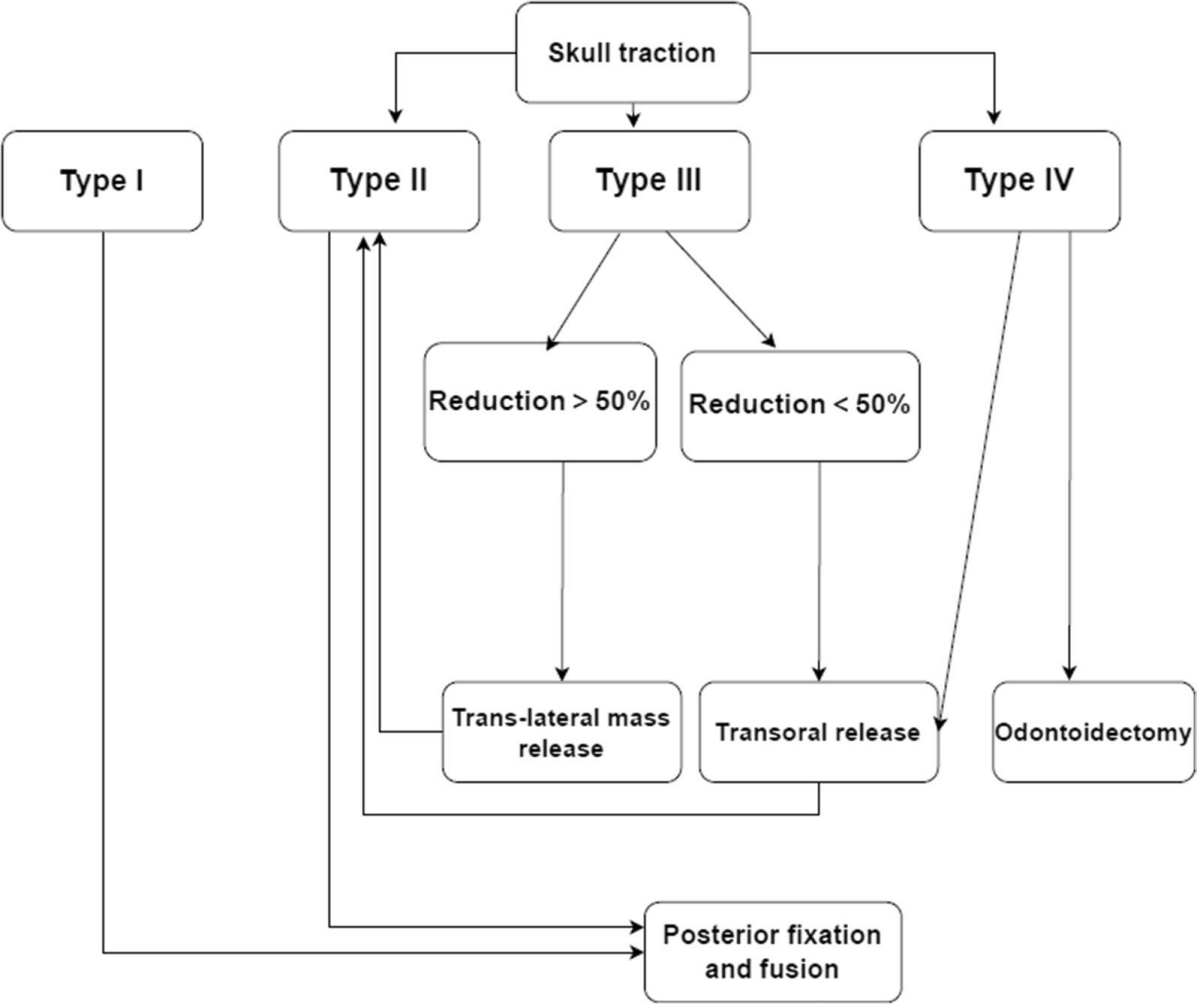
Flow chart of surgical strategies for OO with AAD

The therapeutic regimen (posterior fixation and fusion) is the same for type I and type II AAD. According to one study [10], it seems that irreducible (type III) AAD originates from the unstable atlantoaxial joint. Specifically, the muscles, ligaments, and joint capsules become shorter and eventually contract due to the gradual remodeling of the lateral mass and facet joint, resulting in irreducible AAD. Therefore, attempts to adopt the strategy of posterior trans lateral mass release for type III AAD cases in this study was a right choice, which was also verified by the result that satisfactory reduction was achieved in nine cases after trans lateral mass release. Small changes in the atlanto-odontoid interspace after traction are often complicated with anterior soft-tissue contracture, so this condition should not be treated only by simple trans lateral mass release during operation, making transoral release necessary (performed in five cases in this study). Transoral plate fixation and fusion are also available for type III AAD, but this procedure often has complications such as wound infection, cerebrospinal fluid leakage, nerve injury, and internal fixation loosening [22, 23], thus restricting its popularization, and it was not done in this study. In summary, the recommended operation for type III is posterior fixation and fusion following conversion to type II by trans lateral mass release or transoral release. The possible indications for trans lateral mass release in type III AAD are as follows: (1) a high degree of reduction (> 50%) after skull traction and (2) lateral mass fusion or partial fusion seen on preoperative CT. A low degree of reduction (< 50%) after traction is considered an indication for transoral release (Fig. 7).

In this study, two cases of type IV AAD were converted to type II through transoral bony decompression and release, followed by posterior fixation and fusion. This experience suggests that odontoidectomy alone is not the only approach for type IV. Odontoidectomy may be required if reduction fails following transoral bony decompression and release.

An overview of the surgical strategies for OO with AAD is shown in Fig. 7.

### Efficacy

In the study by Goel et al. [24], all patients showed symptomatic and clinical neurologic recovery. Other studies also obtained satisfactory efficacy. In this study, the JOA score increased from 9.58 ± 1.84 points before operation to 13.09 ± 2.68 points at 3 months after operation, 14.07 ± 2.83 points at 6 months and 14.25 ± 2.34 at 12 months after operation, all statistically significant improvements over the score before operation. The neurological recovery was obvious within 3 months after operation, and it was relatively slow at 3–6 months after operation. It can be seen that 6 months after operation seems to be the time when a plateau in recovery is reached, indicating that maximal rehabilitation within 3 months after operation is a key point. However, there were three patients whose had further neurological deterioration within 6 months after operation, which is rarely reported in the literature. One possible reason is related to the course of disease, atrophy of the spinal cord, and high T2 signals, which also support our previous findings [25] Preoperative kinematic MRI may provide guidance for these patients in determining whether there is a need to resect the posterior arch of atlas during operation [26]. Based on the above three cases, it is plausible that early intervention should also be performed on atlantoaxial instability patients without neurological dysfunction.

### Prevention of complications

In this study, preoperative CT angiography (CTA) showed that the vertebral artery slightly deviated from the midline anteriorly in one case, and the head was tilted slightly to one side in the supine position during operation, causing vertebral artery injury when the lateral mass joint capsule was incised. Despite the active trans arterial embolization later, the patient died of multiple-organ failure. Therefore, it should be noted that preoperative CTA of cervical vessels is recommended, and it is necessary to carefully evaluate for abnormal vessel courses. We also recommend to pay constant attention to the presence or absence of body position changes during the operation.

Postoperative atlantoaxial infection can be catastrophic. Internal fixation can be retained in acute infection following debridement, drainage and anti-infection. In this study, one patient with infection complained of postoperative long-term post-occipital pain, which was misdiagnosed as occipital nerve neuralgia (Fig. 6). Obvious symptoms and imaging manifestations of infections were not found until 2 months after operation, at which time the internal fixation became loosened, so it was necessary to remove the internal fixation. To maintain the atlantoaxial stability, the head, neck, and cervical braces were fixed after debridement. Following infection control, nonroutine occipitocervical fusion with external fixation was performed due to a high-riding vertebral artery and destruction of the original screw track. The management of atlantoaxial infection, although rarely seen, is quite intractable, and individualized strategies are required.

### Limitations

Among the 56 cases enrolled, type I and type II (40 cases) were the majority, so the anterior atlanto-odontoid interspace was almost 0 mm after postoperative reduction and fixation, so it was not compared with preoperative imaging data. Similarly, the cervicomedullary angle was mostly normal before operation, so it was not included in our analyses. These are some limitations of this study. Other limitations were its retrospective nature, its small sample size, and its lack of control group.

## Conclusion

OO with AAD is mostly reducible and can be treated with simple posterior atlantoaxial fixation and fusion, but occipitocervical fusion is rarely performed. The existing classification systems used for guiding surgical strategies should be further improved. Translateral mass release or transoral release combined with posterior fixation and fusion can be adopted for treating irreducible AAD, while transoral release combined with posterior fixation and fusion can be used for management of some patients with bony fusion. A long course of disease, obvious atrophy of spinal cord on T1-weighted images, and high signal intensity of the spinal cord on T2-weighted images before operation may indicate a poor prognosis. Early intervention is also recommended, even for atlantoaxial instability patients without neurological symptoms.

## Data Availability

The authors declare that the databases, application/tool, etc. described in the manuscript are available for testing. **Ethics approval and consent to participate.** The study was in accordance with the Declaration of Helsinki, and was approved by Ethics Committee of The Second Affiliated Hospital of Xi’an Jiaotong University (Xi’an, China). Written consent to participate in this study was obtained from the participants. **Consent for publication.** As to the information and images of the individuals, consent to publish was obtained.

## Abbreviations

OO: Os odontoideum
AAD: Atlantoaxial dislocation
CT: Computed tomography
JOA: Japanese Orthopaedic Association
MRI: Magnetic resonance imaging
